# Repeatability of RRate measurements in children during triage in two Ugandan hospitals

**DOI:** 10.1371/journal.pgph.0003097

**Published:** 2025-01-07

**Authors:** Ahmad Asdo, Alishah Mawji, Isaac Omara, Ivan Aine Aye Ishebukara, Clare Komugisha, Stefanie K. Novakowski, Yashodani Pillay, Matthew O. Wiens, Samuel Akech, Florence Oyella, Abner Tagoola, Niranjan Kissoon, John Mark Ansermino, Dustin Dunsmuir

**Affiliations:** 1 Department of Anesthesiology, Pharmacology & Therapeutics, University of British Columbia, Vancouver, British Columbia, Canada; 2 Institute for Global Health, BC Children’s Hospital and BC Women’s Hospital + Health Centre, Vancouver, British Columbia, Canada; 3 Department of Pediatrics, Gulu Regional Referral Hospital, Gulu, Uganda; 4 WALIMU, Kampala, Uganda; 5 KEMRI-Wellcome Trust Research Programme, Nairobi, Kenya; 6 Department of Pediatrics, Jinja Regional Referral Hospital, Jinja, Uganda; 7 Department of Pediatrics, University of British Columbia, Vancouver, British Columbia, Canada; University of California San Francisco, UNITED STATES OF AMERICA

## Abstract

Pneumonia is the leading cause of death in children globally. In low- and middle-income countries (LMICs) pneumonia diagnosis relies on accurate assessment of respiratory rate, which can be unreliable when completed by nurses with less-advanced training. To inform more accurate measurements, we investigate the repeatability of the RRate app used by nurses in Ugandan district hospitals. This secondary analysis included 3,679 children aged 0–5 years. The dataset had two sequential measurements of respiratory rate collected by 14 nurses using the RRate app. We measured agreement between respiratory rate observations while indicating observations’ clustering around WHO fast-breathing thresholds. WHO thresholds are 60 breaths per minute (bpm) for under two months (Age-1), 50 bpm for two to 12 months (Age-2), and 40 bpm for 12.1 to 60 months (Age-3). We assessed the repeatability of the paired measurements per user through the Intraclass Correlation Coefficient (ICC) and calculated an overall ICC value. The respiratory rate measurement took less than 15 seconds for 7,277 (98.9%) of the measurements. Despite respiratory rates clustering around WHO thresholds, breathing classification based on the thresholds (Fast vs normal) was altered between sequential measurements in only 12.6% of children. The mean (SD) respiratory rate by age group was 60 (13.1) bpm for Age-1, 49 (11.9) bpm for Age-2, and 38 (10.1) for Age-3, and the bias (Limits of Agreements) were 0.3 (−10.8–11.3) bpm, 0.4 (−8.5–9.3) bpm, and 0.1 (−6.8, 7.0) bpm for Age-1, Age-2, and Age-3 respectively. The repeatability of the paired respiratory rate measurements was high, with an ICC ≥ 90% for 12 of 14 users and an overall ICC value (95% CI) of 0.95 (0.94–0.95). The RRate measurements were efficient and repeatable. The simplicity, repeatability, and efficiency support its usage in LMICs healthcare facilities, and endorses a more widespread clinical adoption.

## Introduction

Pneumonia remains the leading infectious cause of death among children under five globally, taking approximately 725,000 young lives annually. These deaths account for 14% of all child mortality, including around 190,000 newborns [1,2]. African regions account for 30% of the global burden of pneumonia [3]. Many of these pneumonia deaths are preventable with accurate diagnosis and prompt treatment.

The World Health Organization (WHO) Integrated Management of Childhood Illness (IMCI) guidelines rely significantly on clinical respiratory rate measurement for diagnosing and managing pneumonia in Low- and Middle-Income Countries (LMICs). However, respiratory rate remains difficult to measure accurately despite its profound clinical importance [4]. First level sick-child support in LMICs is primarily done by nurses with no routine access to more sophisticated diagnostic tools [5]. Nurses in these settings have been shown to make less-sensitive identifications of pneumonia compared to clinicians, suggesting that differences in training might influence diagnostic abilities [6].

The “true” underlying physiological respiratory rate is ephemeral and time-varying; however, one factor that can be measured and optimized in clinical practice is repeatability. Repeatability is the consistency between different sets of measurements taken under similar conditions [7]. Repeatability is independent of the measurement’s “true value.” Repeatability does not guarantee accuracy, but good accuracy must have acceptable repeatability (Table 1) [8]. The uncertainty in respiratory rate measurement introduced by poor repeatability will significantly reduce the reliability of clinical decisions [9]. However, a simple, efficient, and repeatable method of measuring respiratory rate is likely to improve the accuracy of respiratory rate measurements by nurses in LMICs.

**Table 1:** Definitions^a^ and comments on accuracy and repeatability.

Term	Definition
Accuracy	Closeness of the agreement between the result of a measurement and a true value of the measurand
Repeatability	Closeness of the agreement between the results of successive measurements of the same measurand carried out under the same conditions of measurement

^a^Definitions are derived from Joint Committee for Guides in Metrology (JCGM).

The WHO recommends the Acute Respiratory Infection (ARI) timer for measuring respiratory rate [10]. Unfortunately, the usability of the ARI is suboptimal when caring for a restless child who may be moving, crying, or breathing rapidly [11]. In addition, it takes a minimum of a full minute to measure RR with the ARI timer, which is difficult in a busy understaffed clinic or emergency department [11]. RRate is a smartphone application for measuring respiratory rate in LMIC hospital outpatient departments. Previous research has optimized the trade-off between usability and accuracy and compared the RRate app to the ARI timer in a controlled setting [12,13]. The objective of this study was to evaluate the repeatability of RRate when used to measure respiratory rate in a busy outpatient department of a Ugandan hospital. We have previously investigated repeatability of the pulse oximetry measurements taken during this same study [14].

## Methods

Data for this secondary analysis was collected during the baseline phase of a multisite implementation study for a digital triaging platform, Smart Triage (Clinical Trials.gov Identifier: NCT04304235) [15,16]. Measurements were obtained from 4,604 children who presented to the outpatient department of Jinja Regional Referral Hospital (JRRH, 1,748 children) and Gulu Regional Referral Hospital (GRRH, 2,856 children) in Uganda from April, 27^th^ 2020, to April 16^th^ 2022. JRRH and GRRH are both public hospitals in an LMIC and have admission rates of 20% and 18%, respectively. Eligible patients for the original study were those under 19 years of age seeking assessment for an acute illness at the pediatric emergency department between 8:00 am and 5:00 pm. Patients were not eligible if they were there for elective procedures, scheduled appointments or treatment of chronic illnesses [15]. Measurements were performed by nurses employed specifically for this study who were trained in the use of the RRate application during a week-long training of all study procedures. All of the trained nurses had worked previously in a hospital and were from the region where the hospital was located, so they were fluent in the local dialect. Piloting of all training tools was done to ensure high quality data collection once the study began.

RRate requires a minimum of five taps on the screen (i.e., four interbreath intervals) to measure respiratory rate. If the first five taps were inconsistent, the app will continue recording up to 12 taps until a consistency threshold is met for 5 consecutive taps. If this was not met, the app will prompt the user to redo the measurement [13].

### Data collection

After consent, a nurse collected over 200 variables, including clinical signs, symptoms, and sociodemographic variables using a custom data collection app on Android Samsung Galaxy tablets [15]. Data was collected by trained dedicated study nurses while patients were waiting to be triaged by the regular hospital staff. For each child, two measurements of respiratory rate were taken using a version of the RRate app embedded into the data collection app. This version did not allow changes to any settings and used the default RRate settings that balance accuracy and efficiency as determined by a previous study [13]. The user observes the patient’s chest and taps the touch screen on the onset of each inhalation. Inter-breath intervals are then calculated from the time between taps. The RRate app ensures the user taps consistently five times [12].

### Data analysis

This secondary analysis only included children up to 5 years of age. We excluded children without both respiratory rate measurements, those whose paired respiratory rate measurements that were more than five minutes apart, or those who had more than 80% of their clinical data missing. We used Bland Altman plots to assess systematic errors as well as bias and limits of agreement between the first respiratory rate measurement (RR-1) and the second respiratory rate measurement (RR-2). We investigated the respiratory rates clustering around WHO fast breathing thresholds by classifying respiratory rates as normal vs fast and assessing the consistency of this classification between the paired measurements [10]. The thresholds for fast breathing are 60 bpm for children younger than two months (Age-1), 50 bpm for children between two to 12 months (Age-2) and 40 bpm for those between 12.1 to 60 months (Age-3). Additionally, admission rates were compared between age groups using Analysis of Variation (ANOVA).

The Intraclass Correlation Coefficient (ICC) was assessed as a measure of repeatability between RR-1 and RR-2. ICC values have been defined as follows: less than 0.2—slight repeatability, between 0.2 and 0.4—low repeatability, between 0.4 and 0.7—moderate repeatability, between 0.7 and 0.9—high repeatability, and greater than 0.9—very high repeatability [17]. We first calculated an ICC for each observer (healthcare worker). We then calculated the ICC using all measurement pairs with a multiple-rater model to account for any probabilistic dependency between the observers.

### Ethical considerations

The parent study was approved by the institutional review boards at the University of British Columbia in Canada (ID: H19-02398; H20-00484), the Makerere University School of Public Health in Uganda (ID: 743) and the Uganda National Council for Science and Technology (ID: HS528ES). All participants consented to use of their data for secondary analyses. A parent or guardian provided written informed consent prior to enrollment. Data for the retrospective study was accessed on January 27, 2022, and there was no access to identifying patient data.

## Results

**Fig 1:**
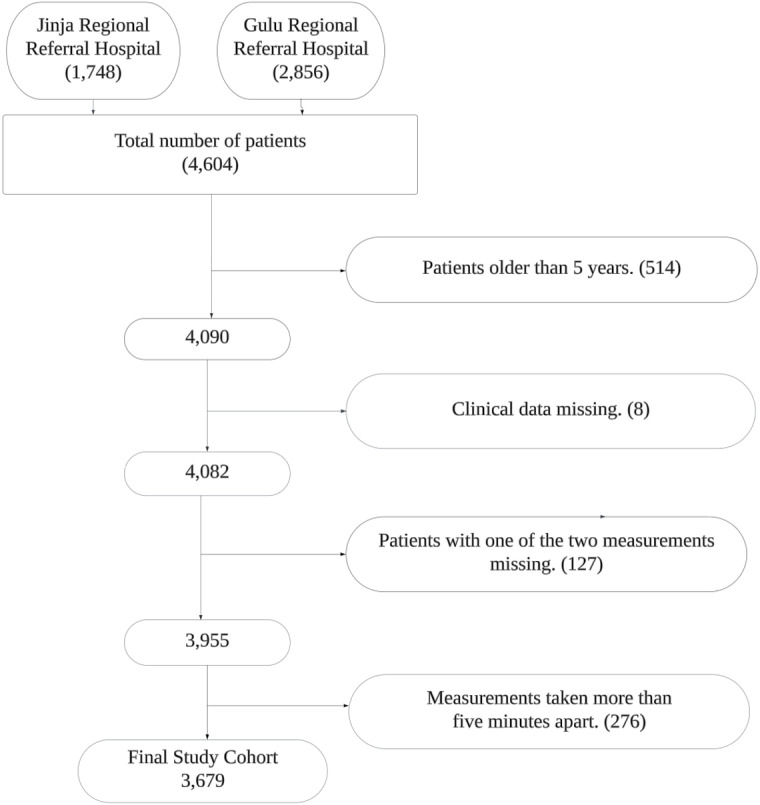
Consort diagram for children included in the study.

We reviewed 4,604 children from the two facilities and excluded 925 who did not meet the eligibility criteria. A total of 3,679 paired observations were analyzed (Fig 1). The median (IQR) time difference between the two respiratory rate measurements was 57 (25–91) seconds. There were 455 patients admitted from the 3,679. The most prevalent diagnoses of admitted patients were malaria, then sepsis, then pneumonia.

The mean (SD) respiratory rate was 44 (12.9) bpm for all children. The mean respiratory rate by age group was within two breaths of the corresponding WHO threshold for fast breathing (Table 2). The admission rate was lower for Age-2 children (Table 2 and S1 Appendix).

**Table 2:** Demographics and parameters of respiratory rate for the study cohort.

Age group	WHO Threshold for fast breathing in bpm	Male sex (N)	Age in months Median (Interquartile range)	RR in bpm Mean (Standard Deviation (SD))	Admission rate (95% Confidence interval))
Age-1	60	58% (149)	1.1 (0.6–1.6)	60 (13.1)	18% (14–23)
Age-2	50	51% (730)	6.8 (4.7–9.4)	49 (11.9)	10% (9–12)
Age-3	40	50% (998)	24.2 (16.7–36.8)	38 (10.1)	14% (12–15)

Study nurses were able to measure respiratory rates from the first 5 breaths (5 taps on the screen, 4 inter-breath intervals) in 6,189 (84.1%) of measurements. For 6,909 (93.9%) measurements, the nurses required 6 or less breaths (6 or less taps) for measurement. A respiratory rate measurement was obtained in less than 15 seconds in 7,277 (98.9%) of the total measurements completed.

**Fig 2:**
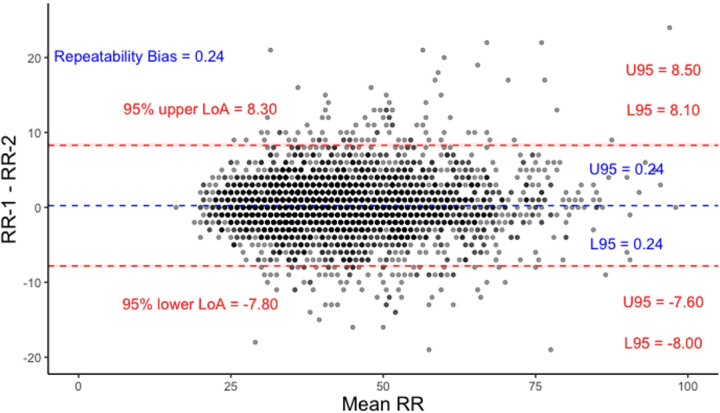
Bland Altman plot for RR-1 and RR-2 across all children. Limits of Agreement (LoAs) and bias are under repeatability conditions, considering one set of samples as the test and the other as the reference. U95 and L95 represent the upper and lower bounds of the 95% CI of the limits of agreements respectively.

A Bland Altman plot for all the children showed strong agreement between RR-1 and RR-2 with a bias of 0.24 breaths per minute (Fig 2).

**Fig 3:**
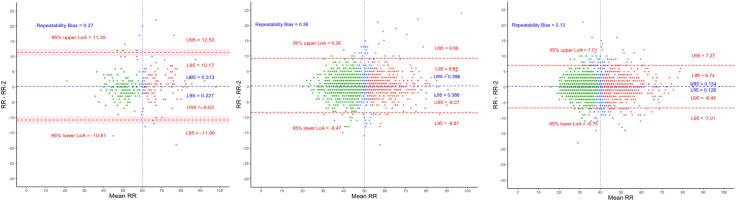
Bland Altman plots for Age-1, Age-2 and Age-3 (from left to right). Threshold for fast breathing is 60, 50 and 40 bpm respectively. Red dots represent children with two fast breathing measurements, green dots – two normal breathing, and blue dots – one fast and one normal. U95 and L95 represent the upper and lower bounds of the 95% CI of the limits of agreements respectively.

The limits of agreements were the widest in Age-1 (−10.81–11.35) bpm and narrowed with increasing patient age with Age-2 LoAs as (−8.47–9.26) bpm and Age-3 (−6.75–7.01) bpm. Additionally, bias was lowest in Age-3 (Fig 3). There were 463 children (12.6%) who had their respiratory rate classification changed according to IMCI (Table 3).

**Table 3:** Children’s breathing classification based on age group and WHO thresholds.

	Percentage of Age-1	Percentage of Age-2	Percentage of Age-3	All children
Two Normal Measurements	46.7%	51.3%	57.0%	54%
Two Fast Measurements	40.5%	35.4%	30.9%	33.4%
One Normal	12.7%	13.3%	12.0%	12.6%

The repeatability performance between RR-1 and RR-2, indicated by the ICC, was ≥0.90 for 12 of 14 users despite the variable sample size measured by each user (Table 4). The overall ICC (IQR) of the two respiratory rate measurements using the interrater model was 0.95 (0.94–0.95). This indicates the very high repeatability of the RRate app.

**Table 4:** Intraclass Correlation Coefficient (ICC) per user.

User	N	ICC	95% CI
1	236	0.96	(0.95–0.97)
2	242	0.98	(0.97–0.98)
3	2	0.99	(0.69–0.99)
4	603	0.93	(0.91–0.94)
5	30	0.99	(0.98–0.99)
6	173	0.98	(0.97–0.99)
7	529	0.88	(0.85–0.89)
8	46	0.97	(0.94–0.98)
9	58	0.96	(0.93–0.98)
10	219	0.93	(0.91–0.95)
11	303	0.96	(0.96–0.97)
12	623	0.95	(0.94–0.96)
13	39	0.89	(0.80–0.94)
14	576	0.98	(0.97–0.98)

## Discussion

In this secondary analysis we found a high repeatability of respiratory rate measurements taken by nurses using the RRate application on children arriving to two busy public referral hospitals in Uganda. The Bland Altman plot (Fig 2) further indicates the strong agreement between the two measurements with a bias (LoAs) of only 0.24 (−7.8–8.3) bpm. Additionally, the app measurements were completed rapidly, as 98.9% of the measurements took less than 15 seconds.

The RRate app is more efficient in LMICs settings compared to ARI timer. While both measuring devices were previously shown to have similar accuracy in a controlled setting where de-identified videos of anesthetized children were used as a standard [13], RRate provides a respiratory rate in children in 15 seconds versus the 60 seconds required using the ARI timer. This is a distinct advantage in settings where healthcare workers need to efficiently and rapidly triage large numbers of children [18]. During the measurement, RRate does not require the child to lie motionless. Another attractive feature of the RR app is its interoperability with an array of mobile devices that can run this application at no additional cost.

A previous study has shown a lack of accuracy in the RRate app when using a video-based counting method as a reference. However, there was no formal training in the use of RRate and repeatability was not reported [19] Those findings were in contrast to another analysis, where RRate showed excellent agreement with the video-based counting reference [12].

Evaluating respiratory rate measurement in experimental controlled settings may not be comparable to real-world settings. However, the demonstration of robust repeatability would be highly desirable for real world applications. Automated respiratory rate devices such as Masimo Rad G pulse oximeter have also been evaluated in real-world settings and have been shown to have variable accuracy with wide 95% LoAs (−34–6) bpm. However, automated device repeatability has not been demonstrated [20].

## Implications for clinical care

Despite the importance of respiratory rate measurement, measuring an accurate rate is an ongoing challenge to healthcare workers [4]. Respiratory rate assessment is relied upon to make clinical decisions in LMICs where the burden of pneumonia is highest and where nurses are heavily relied upon to identify pneumonia. Considering the relatively lower sensitivity of decisions made by nurses compared to those made by clinicians, utilizing the RRate mobile app can enhance the capabilities of nurses who heavily depend on respiratory rate to identify rapid breathing [6]. It will also inform decisions to implement pneumonia treatment protocols. The low cost and accessibility of RRate makes it appropriate for use in low-resource settings, especially when there are many children and few healthcare workers.

## Limitations

While repeatability was assessed, the study did not measure RRate accuracy in real-world clinical settings. The absence of such data limits conclusions about how the device would perform under actual clinical conditions. It is important to recognize that repeatability and accuracy are distinct concepts. While the device may produce consistent measurements, this does not necessarily imply that those measurements are accurate. Without comparison to a gold standard, the accuracy of the device remains uncertain. Furthermore, the study does not explore the clinical significance of the observed differences in measurements. The study also lacks information on the acceptability of the device. The generalizability of these results may be limited since the data is from only two institutions and only includes 14 observers. The time needed to measure RRate is reported starting from when the first breath is observed (tapped). This does not include the time taken to prepare for the measurement, which may include revealing the child’s chest or calming them. However, using a traditional method of counting breaths would also not start until after a child was calmed. In situations where the user failed to tap the screen consistently for 12 breaths, the app would prompt the user to redo the measurement and would not record the time taken for the failed measurement, which might lengthen the time needed to obtain a measurement. We do not have data on how often users failed; however, given that 84.1% of measurements needed only the initial five breaths, and 93.9% needed a maximum of six breaths, it is likely that very few (if any) children needed more than one attempt.

## Conclusion

The RRate app is an open-source and free solution to respiratory rate measurement with very high repeatability and agreement between measurements. RRate is an efficient and repeatable alternative to the breath counting method. The screen tapping method could be incorporated into medical monitoring devices to assess respiratory rate.

## Supporting information

S1 Appendix Differences in admission rates between Age-1, Age-2 and Age-3. (DOCX)

S1 Checklist Human participants research checklist. (DOCX)
