# Supplementary material for: Repeatability of RRate measurements in children during triage in two Ugandan hospitals
Source: PLOS Glob Public Health. 2025 Jan 7;5(1):e0003097. doi: 10.1371/journal.pgph.0003097 (PMC11706398; doi:10.1371/journal.pgph.0003097)
Supplement: S1 Appendix — (DOCX) [file pgph.0003097.s001.docx]

**Appendix:**

Age-2 had significantly lower admission rates compared to Age-1 and Age-3 using Tukey-Kramer test (Table 1A).

|  | **Difference in Admission Rate** | **Lower Bound of 95% CI of Difference** | **Upper Bound of 95% CI of Difference** | **p value** |
| --- | --- | --- | --- | --- |
| Age-2 - Age-1 | -8.1% | -13.2% | -2.9% | 0.0005 |
| Age-3 - Age-1 | -4.9% | -9.9% | 0% | 0.05 |
| Age-3 - Age-2 | 3.1% | 0.005% | 5.8% | 0.015 |

*Table 1A. The difference in admission rates (Tukey-Kramer)*
